# Fine Mapping of a Resistance Gene *RpsHN* that Controls *Phytophthora sojae* Using Recombinant Inbred Lines and Secondary Populations

**DOI:** 10.3389/fpls.2017.00538

**Published:** 2017-04-11

**Authors:** Jingping Niu, Na Guo, Jutao Sun, Lihong Li, Yongce Cao, Shuguang Li, Jianli Huang, Jinming Zhao, Tuanjie Zhao, Han Xing

**Affiliations:** National Center for Soybean Improvement, Key Laboratory of Biology and Genetics and Breeding for Soybean, Ministry of Agriculture, State Key Laboratory of Crop Genetics and Germplasm Enhancement, Nanjing Agricultural UniversityNanjing, China

**Keywords:** soybean, Phytophthora root rot, fine mapping, resistance gene, bins and SSR markers, linkage map

## Abstract

Phytophthora root rot (PRR), caused by *Phytophthora sojae*, has negative effects on soybean yield in China and can be controlled by identifying germplasm resources with resistance genes. In this study, the resistance locus *RpsHN* in the soybean line Meng8206 was mapped using two mapping populations. Initial mapping was realized using two recombinant inbred line (RIL) populations and included 103 F_6:8_ RILs derived from a cross of Meng8206 × Linhedafenqing, including 2600 bin markers, and 130 F_6:8_ RILs derived from a cross of Meng8206 × Zhengyang148, including 2267 bin markers. Subsequently, a 159 F_2:3_ secondary population derived from a cross of Meng8206 × Linmeng6-46, were used to fine map this locus using SSR markers. Finally, the resistance locus from Meng8206 was fine mapped to a 278.7 kb genomic region flanked by SSR markers SSRSOYN-25 and SSRSOYN-44 at a genetic distance of 1.6 and 1.0 cM on chromosome 3 (Chr. 03). Real-time RT-PCR analysis of the possible candidate genes showed that three genes (*Glyma.03g04260, Glyma.03g04300*, and *Glyma.03g04340*) are likely involved in PRR resistance. These results will serve as a basis for cloning, transferring of resistant genes and breeding of *P. sojae*-resistant soybean cultivars through marker-assisted selection.

## Introduction

Phytophthora root rot (PRR), caused by the soil-borne oomycete pathogen *Phytophthora sojae*, is the second highest yield-suppressing disease (Schmitthenner, 1985; Wrather and Koenning, 2009). This disease has resulted in significant economic losses worldwide (Tyler, 2007). In Heilongjiang Province of China, *P. sojae* is widespread but unevenly distributed. It has been estimated that more than 150,000 ha of the soybean grown in fields become infected annually (Tian et al., 2016).

Cultivating *Phytophthora*-resistant soybean cultivars can reduce the incidence of PRR. There are two types of resistant cultivars: race-specific resistance and partial resistance (Sugimoto et al., 2012). Partial resistance is controlled by multiple genes and exhibits broad-spectrum and durable resistance to a range of pathogen species (Kou and Wang, 2010). However, in plants, partial resistance may be lost under high disease pressure to *P. sojae* (Dorrance, 2003). One of the most effective methods to control plant diseases is the development of varieties with vertical resistance genes (Flor, 1971).

To our knowledge, 22 race-specific resistance soybean cultivars containing single genes have been identified and reported: L88-8470 (*Rps1a*), L77-1863 (*Rps1b*), L75-3735 (*Rps1c*), L93-3312 (*Rps1d*), L77-1794 (*Rps1k*), L76-1988 (*Rps2*), L83-570 (*Rps3a*), L91-8347 (*Rps3b*), L92-7857 (*Rps3c*), L85-2352 (*Rps4*), L85-3059 (*Rps5*), L89-1581 (*Rps6*), L93-3258 (*Rps7*), PI 399073 (*Rps8*), Ludou4 (*Rps9*), Wandou15 (*Rps10*), PI 594527 (*Rps11*), PI 567139B (*RpsUN1* and *RpsUN2*), Yudou25 (*RpsYu25*), Yudou29 (*RpsYD29*), Waseshiroge (unnamed *Rps* gene), and Nannong 10-1 (*RpsJS*) (Sugimoto et al., 2011, 2012; Sun et al., 2011, 2014; Wu et al., 2011; Zhang et al., 2013a,b; Ping et al., 2015; Li et al., 2016). Twenty-three genes/alleles identified in the soybean cultivars listed above were located on six chromosomes. The genes/alleles *Rps1* (including five alleles *Rps1-a, Rps1-b, Rps1-c, Rps1-d*, and *Rps1-k*), *Rps7, Rps9, RpsUN1, RpsYu25* and an unnamed *Rps* gene (*Rps1?*) on chromosome 3, *Rps3* (including three alleles *Rps3a, Rps3b, Rps3c*) and *Rps8* on chromosome 13, *Rps2* and *RpsUN2* on chromosome 16, and *Rps4, Rps5* and *Rps6* on chromosome 18 were detected by linkage analysis and genetic mapping (Kilen et al., 1974; Mueller et al., 1978; Athow et al., 1980; Ploper et al., 1985; Buzzell and Anderson, 1992; Cregan et al., 1999; Gordon et al., 2006; Sugimoto et al., 2011; Sun et al., 2011; Wu et al., 2011). *RpsYD29* was mapped to a 204.8-kb region on chromosome 3, and two nucleotide-binding site and leucine-rich repeat (NBS-LRR) type genes *Glyma03g04030.1* and *Glyma03g04080.1* were identified (Zhang et al., 2013b). *Rps1k* has an NBS-LRR structure that is typical of resistance proteins. However, the physical location of *Rps1k* is unknown in the reference genome of ‘Williams 82’ (Gao et al., 2005; Gao and Bhattacharyya, 2008). *RpsJS*, a fine mapping gene located in a 138.9-kb region with 14 candidate genes on chromosome 18, and three genes *Glyma18g51930, Glyma18g51950* and *Glyma18g51960* were characterized as NBS-LRR type genes (Sun et al., 2014). *Rps11* mapped to a 225.3-kb region on chromosome 7, and *Rps10* mapped to a 311-kb region on chromosome 17 (Zhang et al., 2013a; Ping et al., 2015). Furthermore, the *Rps10* mapping region contained two candidate genes, *Glyma17g28950.1* and *Glyma17g28970.1*, annotated as serine/threonine (Ser/Thr) protein kinases.

Another measure for PRR resistance is pyramid-breeding. Pyramiding resistance genes may increase the resistance of soybean cultivars to many pathogen races, and pyramiding genes could be rapidly achieved using molecular markers (Lohnes and Schmitthenner, 1997). Based on the methods of controlling PRR, the identification of a novel *Rps* gene in soybean cultivars is needed to study resistance, and the development of new molecular markers is needed for marker-assisted selection (MAS).

The germplasm Meng8206 (ZDD11436) is a soybean line developed from Yangtze-Huai region of China, studied in drought-tolerance and cyst nematode-tolerance (Duan et al., 2008; Wang et al., 2015). The objectives of the present study were to analyze the inheritance of Meng8206 resistance, identify resistance loci and manipulate predicted candidate genes.

## Materials and Methods

### Plant Materials and *P. Sojae* Isolates

Two F_6:8_ recombinant inbred line (RIL) populations were used for initial mapping: 103 RILs and 130 RILs were constructed from a cross between Meng8206 × Linhedafenqing and Meng8206 × Zhengyang148, respectively. An F_2:3_ secondary population was used for fine mapping: 159 lines were constructed from a cross between Meng8206 × Linmeng6-46 (Supplementary Figure 1). The soybean lines Meng8206, Linhedafenqing, Zhengyang148 and Linmeng6-46 were obtained from National Center for Soybean Improvement, Nanjing Agricultural University, Nanjing, China. Meng8206 was also obtained from the Chinese National Soybean GeneBank (CNSGB).

To clarify the response type of Meng8206 to *P. sojae*, 15 differentials were used, and each cultivar had an independent *Rps* gene. The 15 differential cultivars included Harlon (*Rps1a*), Harosoy13XY (*Rps1b*), Williams79 (*Rps1c*), PI103091 (*Rps1d*), Williams82 (*Rps1k*), L76-1988 (*Rps2*), Chapman (*Rps3a*), PRXI46-36 (*Rps3b*), PRXI 45-48 (*Rps3c*), L85-2352 (*Rps4*), L85-3059 (*Rps5*), Harosoy62XY (*Rps6*), Harosoy (*Rps7*), Yudou25 (*RpsYu25*), and LuDou4 (*Rps9*). In addition, Williams (no known *Rps* gene) was a susceptible variety used as an inoculation reference.

### *P. sojae* Isolates and Disease Evaluation

A total of eight *P. sojae* isolates (Supplementary Table 1) with different virulence capabilities were provided by Professor Yuanchao Wang of Nanjing Agricultural University and maintained on V8 juice agar medium (10% V8 vegetable juice, 0.02% CaCO_3_ and 1.0% Bacto-agar). These isolates were used to evaluate the resistance identified among the parents and 15 differential cultivars. *P. sojae* HeN08-35 (virulence formula is 3a, 3c, 4, 5, 6 and 7) was used to evaluate two mapping populations.

A modified hypocotyl inoculation technique was utilized for disease evaluation in this experiment (Sun et al., 2011, 2014). All materials were planted in plastic pots containing vermiculite; the mycelia from 7-day-old seedlings were maintained on V8 juice agar and subsequently inoculated onto wounded hypocotyls. After inoculation, the seedlings were placed in a high humidity mist chamber for 48 h and subsequently transferred to a greenhouse at 25°C with a 14-h light/10-h dark photoperiod for 5 days. Two F_6:8_ RILs and F_2:3_ family reactions were evaluated at 5 days post-inoculation (DPI) and recorded as the percentage of dead seedlings. Each family had 30 plants scored. The standard criterion of each family is as follows: if the percentage of dead seedlings >80%, then this family was recorded as homozygous susceptible (S); if the percentage of dead seedlings <20%, then this family was recorded as homozygous resistant (R); and if the percentage of dead seedlings is between 21 and 79%, then this family was recorded as heterozygous resistant (Rs) (Gordon et al., 2006; Ping et al., 2015).

### SNP Genotyping and Bin Map Construction

The genomic DNA was extracted from the young leaves of two RIL populations according to Zhang and Wang (2004) and used to construct the genomic DNA library after *Taq* I digestion according to Baird et al. (2008). The 400- to 700-bp DNA fragments were sequenced using the Illumina HiSeq 2000 standard protocol for MSG (multiplexed shotgun genotyping), and 90-mer paired-end reads were generated (Andolfatto et al., 2011). SOAP2 (Li et al., 2009) software was used for aligning the sequenced reads to the Williams 82 reference genome. SNP calling and genotyping were conducted using RealSFS software (Yi et al., 2010) based on the Bayesian estimation. Subsequently, using a three-standard filter, 50 < depth < 2500, a probability of site mutation ≥95%, and every SNP loci separated by at least 5 bp, we obtained high confidence SNPs.

Bin maps were constructed using a sliding window approach. The sliding window contained 15 SNPs. As the window slides, the genotypes are called and recombination breakpoints are determined. The same genotype across the entire RIL population was recognized as a single recombination bin (Huang et al., 2009).

### DNA Preparation of F_2_ Individuals and Pooling for Bulk Segregation Analysis

Plant genomic DNA was extracted from young leaves using the CTAB method with minor modifications (Allen et al., 2006). Resistant and susceptible bulks for the bulk segregation analysis (BSA) were, respectively, formed using the plant genomic DNA of 10 resistant and 10 susceptible F_2_ individuals (Michelmore et al., 1991), and the DNA concentration for the two bulks was greater than 50 ng/μl.

### SSR Marker Development and PCR

According to the initial mapping physical position, the sequence was downloaded from Phytozome Glyma1.0^1^, and simple repeat sequences were assessed using SSR Hunter 1.3^2^. New SSR markers were designed using Primer Premier 5.0 (Premier Biosoft International, Palo Alto, CA, USA). In addition, some markers from Soybase^3^ and the published paper (Zhang et al., 2013b) were used. PCR was conducted according to Sun et al. (2014).

### Data Analysis and Genetic Linkage Analysis

A goodness-of-fit to the Mendelian segregation ratio was calculated using Chi-square (χ^2^) analysis to examine the segregation patterns of the phenotypes and selected SSR markers. The resistance locus for initial mapping was detected using composite interval mapping (CIM) in QTL Cartographer 2.5(threshold value 2.5) (Wang et al., 2012). The linkage map of *RpsHN* for fine mapping was constructed using Joinmap 4.0 linkage analysis software (van Ooijen, 2006). The linkage groups were analyzed with a log-likelihood (LOD) threshold of 3.0.

### Expression Analysis of Candidate Genes

Meng8206 (R) and LinMeng6-46 (S) seedlings were cultivated for 7 days and subsequently inoculated with isolate HeN08-35. Approximately 1-cm samples of the treated hypocotyl tissues were collected at five time points. Total RNA was extracted from the plants using the RNA Simple Total RNA kit (TIANGEN, China). cDNA was synthesized using the Prime Script^TM^ RT Reagent Kit (TaKaRa, Japan) using a standard protocol. The experiment was repeated three times.

The CDS sequences for the candidate genes were obtained from Phytozome^4^. The primers for qRT-PCR were designed using Primer Premier 5.0. In addition, the housekeeping gene *Actin* was used as a control. These primers are shown in Supplementary Table 2, and qRT-PCR was conducted using a Light Cycler 480 instrument.

## Results

### Phenotype Reaction of the Parents to *P. sojae* Isolates

**FIGURE 1:**
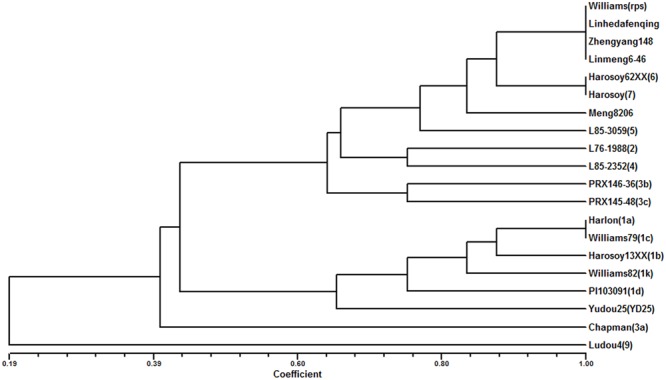
**Dendrogram reflected by UPGMA cluster analysis of the reaction to 8 *P. sojae* among Meng8206, Zhengyang148, Linhedafenqing, and LinMeng6-46, 15 differentials each had an independent *Rps* gene: Harlon (*Rps1a*), Harosoy13XX (*Rps1b*), Williams79 (*Rps1c*), PI103091 (*Rps1d*), Williams82 (*Rps1k*), L76-988 (*Rps2*), Chapman (*Rps3a*), PRX146-36 (*Rps3b*), PRX145-48 (*Rps3c*), L85-2352 (*Rps4*), L85-3059 (*Rps5*), Harosoy62XX (*Rps6*), Harosoy (*Rps7*), YuDou25 (*RpsYu25*), LuDou4 (*Rps9*) and the susceptible cultivar Williams**.

The 8 different isolates were applied to evaluate 4 parents and 15 differentials. The results showed that Meng8206 was resistant to the HeN08-35 isolate and was susceptible to the other 7 isolates (Supplementary Table 3). The phenotype reaction of Meng8206 was different for each of the 15 differentials, conferred through an independent *Rps* gene. The three parents Zhengyang148, Linhedafenqing and Linmeng6-46 were susceptible to all selected *P. sojae* isolates, and their phenotype reactions were the same as Williams. In addition, we also analyzed the genetic diversity and phenotypic relationships of the four parents and 15 differentials to 8 *P. sojae* using cluster tree analysis in the NTSYS program (**Figure 1**). When the coefficient was more than 0.8, Meng8206 existed independently as a subgroup, suggesting that Meng8206 may possess a novel *Rps* gene.

### Inheritance of Resistance to *P. sojae* HeN08-35

In initial mapping populations, among the 103 F_6:8_ RILs derived from a cross of Meng8206 × Linhedafenqing, 48 RILs were homozygous resistant (R), 55 RILs were homozygous susceptible (S) to *P. sojae* isolate HeN08-35, and the actual segregation ratio was consistent with expected ratio 1:1 (χ^2^ = 0.49 and *p* = 0.49) (**Table 1**). This result suggested that Meng8206 resistance was controlled by a single gene. Thus, this locus was temporarily designated *RpsHN*. Among the 130 F_6:8_ RILs derived from a cross of Meng8206 × Zhengyang148, 44 RILs were homozygous resistant (R), and 86 RILs were homozygous susceptible (S) to *P. sojae* HeN08-35. The phenotype data showed no clearly inheritance mechanisms of quality traits. Thus, it was decided to perform QTL analysis for mapping of PRR resistance in this population.

**Table 1:** Segregation analysis of resistance to *P. sojae* HeN08-35 in 103 RILs of Meng8206 × Linhedafenqing.

Parents and RILs individuals	Observed numbers		χ^2^ tests		
	R	S	Expected ratio	χ^2^	*p*
Meng8206(P_1_)	30P	0			
Linhedafenqing (P_2_)	0	30P			
Meng8206 × Linhedafenqing(F_6:8_)	48F	55F	1:1	0.49	0.49

*R, homozygous resistant; S, homozygous susceptible; P, plants number; F, families number.*

In the fine mapping population, among the 159 F_2:3_ individuals derived from a cross of Meng8206 × Linmeng6-46, 38 were homozygous resistant (R), 69 were segregating individuals Rs, 52 were homozygous susceptible (S) to *P. sojae* HeN 08-35, and the actual segregation ratio was consistent with the expected ratio 1:2:1 (χ^2^ = 4.77, *p* = 0.09) (**Table 2**). This result suggested that Meng8206 resistance was controlled by a single dominant gene *RpsHN*.

**Table 2:** Segregation analysis of resistance to *P. sojae* isolates HeN08-35 in 159 F_2:3_ families of Meng8206 × Linmeng6-46.

Parent and cross	Observed numbers			χ^2^ tests		
	R	Rs	S	Expected ratio	χ^2^	*p*
Meng8206(P_1_)	35P	0	0			
Linmeng6-46(P_2_)	0	0	38P			
Meng8206 × Linmeng6-46(F_2:3_)	38F	69F	52F	1:2:1	4.77	0.09

*R, homozygous resistant; Rs, heterozygous resistant; S, homozygous susceptible; P, plants number; F, families number.*

### Initial Mapping of the *RpsHN* Gene

To analyze the resistance locus of Meng8206 in the two RIL populations, a bins map was constructed using a sliding window approach. A total of 2600 bins were identified in the RIL population derived from a cross of Meng8206 × Linhedafenqing; the genetic distance was 2626.00 cM, and the average genetic distance between markers was 1.01 cM. A total of 2267 bins were identified in the RIL population derived from a cross of Meng8206 × Zhengyang148; the genetic distance was 2584.38 cM, and the mean genetic distance between markers was 1.14 cM.

For the Meng8206 × Linhedafenqing RIL population, a resistance locus was detected on Chr03 with an LOD score of 56.89 using CIM. This locus was located between marker bin249 and bin250, at nucleotide positions 3,515,595 and 4,237,477, respectively, determined through a BLAST search in Glyma1.0. The additive effect of this locus was 0.51 and resistant allelic effect came from Meng8206 (Supplementary Figures 2A,B and **Table 3**). Interestingly, only a significant resistance locus with an LOD score of 22.66 was also identified on Chr. 03 in the Meng8206 × Zhengyang148 RILs population, and this locus was located between marker bin282 and bin283, at nucleotide positions 3,564,629 and 4,734,455, respectively. The additive effect of this locus was 0.34 and resistant allelic effect came from Meng8206 (Supplementary Figures 2C,D and **Table 3**). Based on the physical location of the marker, an intersection was detected in the two RILs populations. Because Meng8206 was the same resistance parent in the two RIL populations, the two loci was the same locus.

**Table 3:** QTL mapping of *RpsHN* in the F_6:8_ RILs of Meng8206 × Linhedafenqing and Meng8206 × Zhengyang148.

Population	LG	Position	Marker interval	Physical position	LOD	Variance explained (%)	Additive effect
Meng8206 × Linhedafenqing (F_6:8_)	N	23.1	Bin249–Bin250	3,515,595–4,237,477	56.89	87.2	0.51
Meng8206 × Zhengyang 148(F_6:8_)	N	19.5	Bin282–Bin283	3,564,629–4,734,455	22.66	48.6	0.34

### Fine Mapping of the *RpsHN* Locus

**FIGURE 2:**
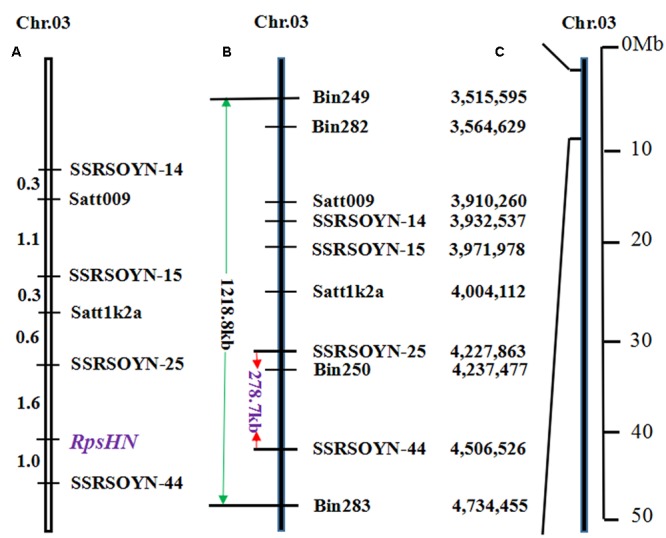
**Genetic linkage map and physical map of *RpsHN* on chromosome 3**. Genetic distance (cM) is shown on the left, and markers are shown on the right. **(A)** Linkage map of *RpsHN* from the present study. **(B)** Physical map of SSR and bins markers from the present study. The green line refers to the physical distance between bin249 and bin283, and the red line refers to the fine mapping region. **(C)** Physical position of the mapped region of *RpsHN* on chromosome 3 (Lin et al., 2013).

We conducted fine mapping on the region between markers bin249 and bin283. The physical distance of bin249 and bin283 is approximately 1218.8 kb (**Figures 2B,C**). In this region, 33 and 6 SSR markers were selected according to Song et al. (2010) and Zhang et al. (2013b), respectively. The two markers satt009 and satt1k2a showed polymorphisms between Meng8206 and Linmeng6-46 using the BSA method.

In addition, a total of 183 repeat motifs (SSR loci) in this region were identified using SSR hunter and used for the fine mapping of *RpsHN*. Four SSR markers, SSRSOYN-14, SSRSOYN-15, SSRSOYN-25 and SSRSOYN-44, showed polymorphisms between Meng8206 and Linmeng6-46 using the BSA method (Supplementary Table 4). Together with satt009 and satt1k2a, six polymorphic marker segregation patterns were revealed by analyzing 159 F_2:3_ families, consistent with the 1:2:1 ratio (Supplementary Table 5).

A genetic map, including six SSR markers and *RpsHN* was constructed, and *RpsHN* was closely linked to the SSR markers SSRSOYN-25 and SSRSOYN-44 at genetic distances of 1.6 and 1.0 cM, respectively (**Figure 2A**).

### Candidate Gene Prediction

The genomic region of Williams 82 was delimited by the markers SSRSOYN-25 and SSRSOYN-44. A BLAST search showed that the physical distance of SSRSOYN-25 and SSRSOYN-44 are at nucleotide position 4,227,863 and 4,506,526 in Glyma1.0, is appropriately 278.7 kb (**Figure 2B**). A total of eight genes were annotated according to the Glyma 1.0 (Supplementary Table 6). Among these genes, *Glyma.03g04260* and *Glyma.03g04300* encoded NB-ARC domain-containing disease resistance protein. *Glyma.03g04340* encodes serine/threonine protein kinase (STK), which is involved in plant disease resistance. These genes were predicted as possible candidate genes.

**FIGURE 3:**
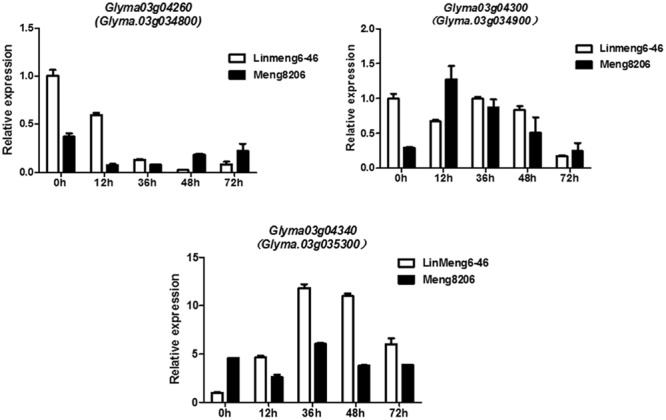
**Relative expression levels of *Glyma.03g04260 (Glyma.03g034800), Glyma.03g04300 (Glyma.03g034900)* and *Glyma.03g04340 (Glyma.03g035300)* in Linmeng6-46 and Meng8206.** Seven-day-old soybean seedlings were inoculated with isolate HeN08-35. The sampling times were 0, 12, 36, 48, and 72 hours post-inoculation (hpi).

To confirm whether *Glyma.03g04260, Glyma.03g04300* and *Glyma.03g04340* were induced under the treatment of *P. sojae*, the expression patterns of three genes were examined using qRT-PCR analysis in Meng8206 and Linmeng6-46. As shown in **Figure 3**, compared with the control (0 h), the expression of *Glyma.03g04260* was down-regulated at 12, 36, and 48 h after treatment in the resistant line Meng8206 and the susceptible line Linmeng6-46, and the expression of *Glyma.03g04300* did not significantly change in Linmeng6-46 and was up-regulated in Meng8206 at 12, 36, and 48 h after treatment. *Glyma.03g04340*, compared with *Glyma.03g04300*, had opposite expression levels. These results showed that three genes were induced by *P. sojae* HeN08-35. Thus, *Glyma.03g04260, Glyma.03g04300* and *Glyma.03g04340* were considered as potential candidate genes.

## Discussion

Soybean [*Glycine max* (L.) Merr.] is one of the most important oil crops in China. Many cultivars/lines have been studied for resistance to *P. sojae* and the identification of resistance loci (Zhang et al., 2014; Huang et al., 2016). In the present study, we identified the loci of the Meng8206 for resistance to *P. sojae* HeN08-35 using two mapping populations. Based on the phenotype reaction types of Meng8206 and the physical position of *RpsHN* on chromosome 3, we inferred that *RpsHN* is a novel gene tightly linked to *Rps1* or a new allele at the *Rps1* locus. Three genes, *Glyma.03g04260, Glyma.03g04300* and *Glyma.03g04340*, were considered as potential candidate genes.

Soybean line Meng8206 was evaluated using eight isolates with different virulence formulas in this study. The results showed that Meng8206 was resistant to the HeN08-35 isolate and was susceptible to the other seven isolates. The parent Meng8206 showed resistance to HeN08-35 isolate as shown in Supplementary Figure 3. We proposed Meng8206 contains at least one novel locus resistant to HeN08-35. Fortunately, we used the F_6:8_ RIL populations derived from a cross of Meng8206 × Linhedafenqing and Meng8206 × Zhengyang148 to map the locus, and found that only a single resistance locus was detected on Chr03. Subsequently, this locus was verified by secondary populations derived from a cross of Meng8206 × Linmeng6-46. To F_6:8_ RIL population (Meng8206 × Zhengyang148), only a significant resistance locus was detected between marker bin282 and bin283 which fitted the expected 1:1 segregation ratio by χ^2^ test. Segregation distortion of phenotype may be due to the variation in genetic background of the progenies.

Previous studies have shown that 11 *Rps* genes were mapped to the N group, *Rps1* allele genes (including five alleles *Rps1a, Rps1b, Rps1c, Rps1d*, and *Rps1k*), *Rps7, Rps9, Rps1?, RpsUN*1 and *RpsYD29*. *RpsHN* clustered in a subgroup different from the *Rps1* alleles, *RpsYu25* and *Rps9* subgroups. *Rps7* was located above marker satt009 (Bernard and Cremeens, 1981; Wu et al., 2011), while *RpsHN* was located below marker satt009; thus, the *RpsHN* locus is different from *Rps7*. The *Rps* gene in Waseshiroge (Sugimoto et al., 2011) was located “below” Satt009 and was flanked by Satt009 (0.9 cM), while *RpsHN* was located “below” Satt009 (3.6 cM) and was located “above” SSRSOYN-44 and was flanked by SSRSOYN-44(1.0 cM). So the *Rps* gene from Waseshiroge may be located close to Satt009 (nucleotide position 3,919,203), and *RpsHN* located close to SSRSOYN-44 (nucleotide position 4,506,526) (**Figure 2A**). Because Willimas82 was acted as reference sequence, *RpsHN* may be different from the *Rps* gene in Waseshiroge. *RpsUN1* (Li et al., 2016) locus mapped between BARCSOYSSR_03_0233 and BARCSOYSSR_03_0246 which were unfortunately not polymorphic between the two parents Meng8206 and Linmeng6-46. Landrace PI 567139B (*RpsUN1*) was resistant to *P. sojae* pmg (17)-1 (pathotypes corresponding to races 17) and Meng8206 (*RpsHN*) was susceptible to *P. sojae* P7063 (pathotypes corresponding to races 17) (Supplementary Table 3), two mapping parents had different resistance reaction. So we think *RpsUN1* may be different from *RpsHN*. *RpsYD29* (Zhang et al., 2013b) and *RpsHN* were separated by the SSR marker satt1k2a. These results indicated that *RpsHN* may be a new gene difference from *Rps1* or a new allele gene of *Rps1*.

Two types of soybean resistance genes to *P. sojae* have successfully been cloned thus far, NBS-LRR types and protein kinases. Four coiled-coil (CC)-NBS-LRR type genes were BAC-cloned in the *Rps1k* fine mapping region (Gao et al., 2005; Gao and Bhattacharyya, 2008). Two serine/threonine protein kinase type genes were cloned in the *Rps10* fine mapping region (Zhang et al., 2013a). In the present study, two NB-ARC type genes and a protein kinase-type gene were considered as potential candidate genes. Three candidate genes *Glyma.03g04260, Glyma.03g04300* and *Glyma.03g04340* can be further studied for resistance pathways and functions.

## Conclusion

We identified putatively a novel resistance gene, *RpsHN*, which can be used for breeding cultivars for *Phytophthora* resistance. The tightly linked SSR markers SSRSOYN-25 and SSRSOYN-44, as the functional markers, could contribute to the MAS breeding program.

## Author Contributions

HX and TZ conceived the research. HX and JN designed the research. JN, NG, JS, LL, YC, SL, JH, and JZ performed the experiments and analyzed the data. JN drafted the manuscript. HX and TZ revised the paper.

## Conflict of Interest Statement

The authors declare that the research was conducted in the absence of any commercial or financial relationships that could be construed as a potential conflict of interest.

## Footnotes

1 http://www.phytozome.net/soybean

2 http://www.bio-soft.net/dna/SSRHunter.htm

3 http://soybase.org

4 http://www.phytozome.net/soybean
