# Supplementary material for: Fine Mapping of a Resistance Gene RpsHN that Controls Phytophthora sojae Using Recombinant Inbred Lines and Secondary Populations
Source: Front Plant Sci. 2017 Apr 11;8:538. doi: 10.3389/fpls.2017.00538 (PMC5387331; doi:10.3389/fpls.2017.00538)
Supplement: Supplementary file 1 [file Data_Sheet_1.pdf]

Supplementary Material

Fine mapping of a resistance gene *RpsHN* that controls *Phytophthora sojae* using recombinant inbred lines and secondary populations

First Author*：Jingping Niu

***Corresponding author**

Han Xing
hanx@njau.edu.cn
Tuanjie Zhao
tjzhao@njau.edu.cn

Supplementary Table 1 Virulence pathotype of 8 *P. sojae* isolates classified using 15 differentials carrying *Rps1a*,*Rps1b*,*Rps1c*,*Rps1d*,*Rps1k*,*Rps2*, *Rps3a*,*Rps3b*,*Rps3c*,*Rps4*, *Rps5*, *Rps6* and *Rps7*, respectively

Isolate	Virulence pathotype
P7063	1a, 1d, 3a, 6, 7
S2	1a, 1b, 1c, 1d, 1k, 3a
PNJ1	1d, 2, 3b, 3c, 4, 6, 7
Pmg	1b, 1d, 2, 3a, 3b, 4, 5, 6, 7
Pm28	1a, 1b, 1c, 1d, 1k, 2, 3b, 3c, 5, 6, 7
HeN08-35	3a, 3c, 4, 5, 6, 7
H15	1k, 3b, 3c, 5, 6, 7
Pm31	1a, 1b, 1c, 1d, 1k, 2, 3b, 3c, 4, 5, 6, 7

Supplementary Table 2 Primers of the candidate genes for real-time PCR

Gene ID	Primer sequence(5'-3')	Length(bp)	Annealing temp. (° C)
Glyma03g04260 *(Glyma.03g034800)*	F:CCATGCGTGTTTTTCCTCCG<br>R:ACTGTGCTGCTAAAGGCAGT	104	59
Glyma03g04300 *(Glyma.03g034900)*	F:TCCGAATCGAACGGGAACACA<br>R:TCCCAATGTCACGCTTTCTTCTCA	127	60
Glyma03g04340 *(Glyma.03g035300)*	F:CTTTGGCCTCGGTTGATTAT<br>R:ACCACTACCACCCTGAGGAG	122	60

Supplementary Table 3 The reaction of 15 differentials to 8 *P.sojae*.

Differentials (*Rps*)	*P. sojae* 7063、S2、PNJ1、Pmg、Pm28、HeN35、H15、Pm31
Williams(*rps*)	SSSSSSSS
Harlon(*Rps1a*)	RSRRSRRS
Harosoy13XY(*Rps1b*)	RSRSSRRS
Williams79(*Rps1c*)	RSRRSRRS
PI103091(*Rps1d*)	RSSSSRRS
Williams82(*Rps1k*)	RSRRSRSS
L76-1988(*Rps2*)	SRSSSRRS
Chapman(*Rps3a*)	SSRSRSRR
PRXI46-36(*Rps3b*)	RRSSSRSS
PRXI45-48(*Rps3c*)	RRSRSSSS
L85-2352(*Rps4*)	SRSSRSRS
L85-3059(*Rps5*)	SRRSSSSS
Harosoy62XY(*Rps6*)	SRSSSSSS
Harosoy(*Rps7*)	SRSSSSSS
Meng8206	SSSSS**R**SS
Linmeng6-46	SSSSSSSS
Yudou25(*RpsYD25*)	-SRRRRRS
Ludou4(*Rps9*)	---RRRS-

R: Resistance；S: Susceptible；“-” : Absence.

Supplementary Table 4 Developing four SSR markers linked to *RpsHN*

Primer[a]	Forward sequence（5’-3’）	Reverse sequence（5’-3’）	Tm(℃)
SSRSOYN-14	AACAACAAGCAGCAAGTGAT	CACCCACTATTCGTGATTCC	55
SSRSOYN-15	AAAGTCTAATGTATATTTCAGGTT	GACCAATAGATGTAATGCCAAT	54
SSRSOYN-25	GCTCGGGTGAAATAATAG	ATTTATGATTAGTTGACAGTG	47
SSRSOYN-44	TTGTCACTACTTTGTCCCATAC	CCTGATAACGCCCTAAACT	55

[a]Markers SSRSOYN-14, SSRSOYN-15, SSRSOYN-25 and SSRSOYN-44 were developed according the sequence between bin249 and bin283 on soybean chromosome 3 in the Phytozome database (http://www.phytozome.net)

Supplementary Table 5 Segregation of 6 selected SSR markers among the 159 $F_{2:3}$ families derived from the cross between Meng8206 and Linmeng6-46

Marker[a]	Observed number[b]			Expected ratio and Goodness of fit		
	R	H	S	Expected ratio	χ^2	*P*
SSRSOYN-14	45	67	47	1:2:1	3.84	0.15
Satt009	43	69	46	1:2:1	2.54	0.28
SSRSOYN-15	41	69	48	1:2:1	3.05	0.22
Satt1k2a	41	70	48	1:2:1	2.78	0.25
SSRSOYN-25	38	73	48	1:2:1	2.25	0.32
SSRSOYN-44	41	65	49	1:2:1	4.77	0.09

[a]Markers SSRSOYN-14, SSRSOYN-15, SSRSOYN-25 and SSRSOYN-44 were developed according to the sequence between bin249 and bin283 on soybean chromosome 3 in the Phytozome database (http://www.phytozome.net); Satt009 was obtained from the published map in the Soybase (http://soybase.org); Satt1k2a was obtained from Zhang et al. (2013b).

[b] *R* SSR allele from the resistant parent, *H* SSR alleles from both resistant and susceptible parents, *S* SSR allele from the susceptible parent.

Supplementary Table 6 Gene annotations of soybean chromosome 3 between 4,227,863 bp and 4,506,526 bp

Gene name Glyma 1.0（Glyma 2.0）	Chromosome location	Gene annotations（Glyma 2.0）
Glyma03g04200（*no find*）	4250529- 4257697	none
Glyma03g04260 (*Glyma.03g034800*)	4313547- 4317969	NB-ARC domain-containing disease resistance protein
Glyma03g04300 (*Glyma.03g034900*)	4389879- 4394642	NB-ARC domain-containing disease resistance protein
Glyma03g04330 (*Glyma.03g035200*)	4456756- 4465340	Zinc finger CW-type coiled-coil domain protein
Glyma03g04340 (*Glyma.03g035300*)	4465554- 4469715	Serine/threonine protein kinase
Glyma03g04350 (*Glyma.03g035400*)	4473801- 4476484	PPR repeat
Glyma03g04360 (*Glyma.03g035500*)	4479873- 4484975	Plant mobile domain
Glyma03g04370 (*Glyma.03g035600*)	4485903- 4487295	Protease inhibitor/seed storage/LTP family

Meng8206 ♂ × Linhedafenqing ♀

↓

Linmeng6-46 (a line from $F_{6:8}$ RILs population)

Supplementary Fig. 1: Pedigree of soybean line Linmeng6-46

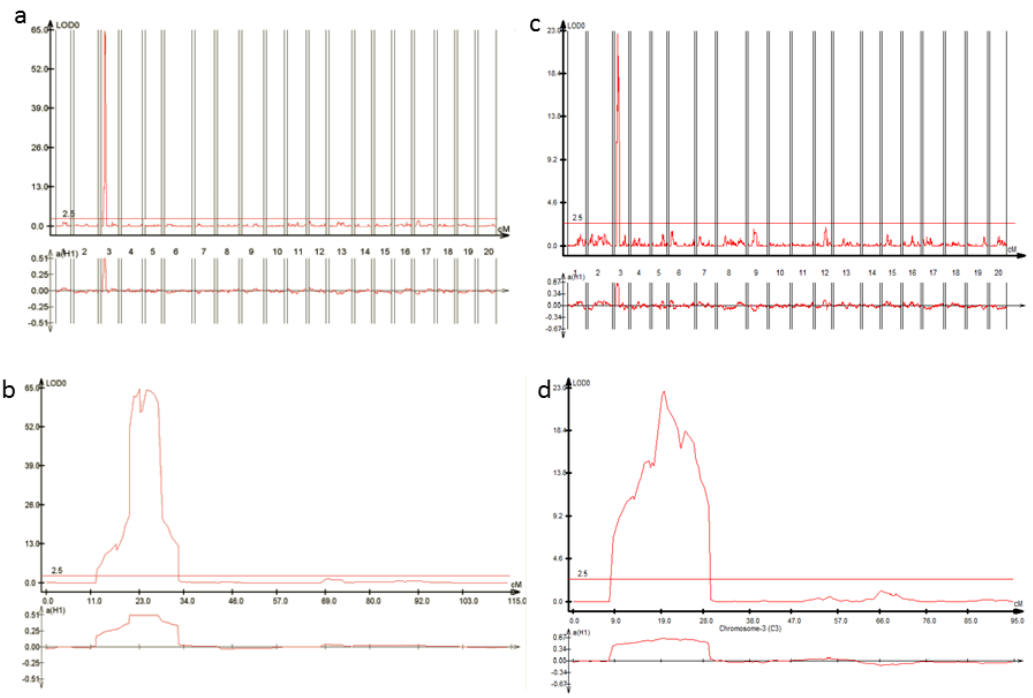

Supplementary Fig. 2 Results of resistance locus analysis using composite Interval Mapping (CIM) method in two RILs. **a** The *LOD* values distribution on the whole genome of Meng8206×Linhedafenqing RILs population. **b** The LOD values distribution on linkage group N(Chr03) of Meng8206×Linhedafenqing RILs population. **c** The *LOD* values distribution on the whole genome of Meng8206×Zhengyang148 RILs population. **d** The LOD values distribution on linkage group N (Chr03) of Meng8206×Zhengyang148 RILs population.

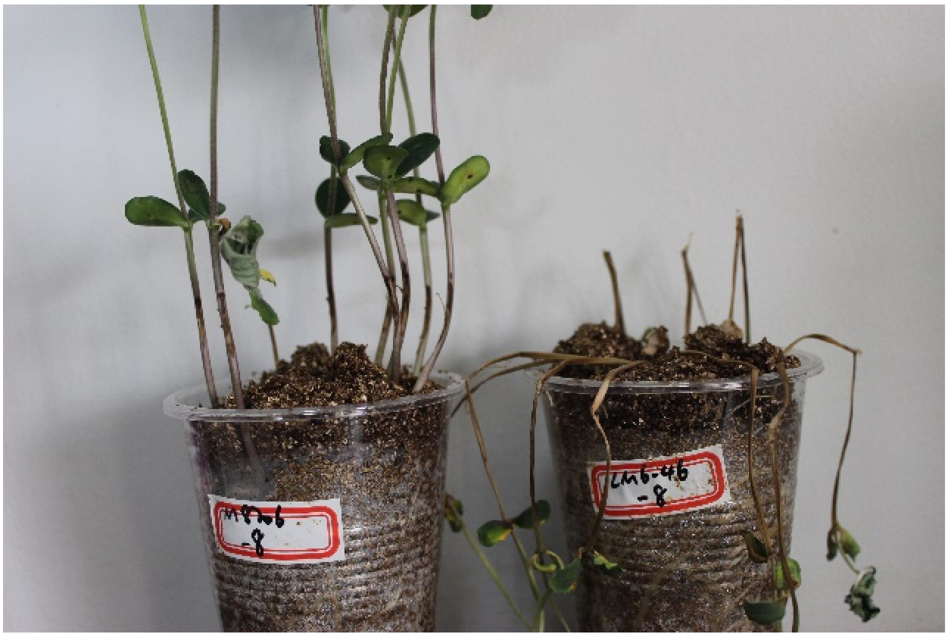

Supplementary Fig. 3 *Phytophthora* resistance test of Meng8206 (left) and Linmeng6-46 (right) inoculated with the *P.sojae* isolate HeN08-35
